# Angling

**Published:** 1875-07

**Authors:** 


### The Bistoury.

ELMIRA, N. Y., JULY, 1875.

The BISTOURY is published Quarterly, upon the 1st of April, July, October and January, at Fifty Cents a year, in advance.

### ANGLING.

Perhaps, nine-tenths of our readers will skip this article after reading its title; but, don't *you* do it, particularly if you are a professional man, and accustomed to use your brain more than your muscle. Lawyers, doctors, clergymen, teachers, editors and shop-keepers, are a few of the professions and occupations that exhaust the nervous and enervate the muscular systems, causing many of the ills for which the physician is consulted during the summer months. To persist in these sedentary occupations year after year, with the sole intent of adding money to your purse, or increased knowledge to one's profession, is frightfully injurious to health and intellect. In what manner this waste is brought about, we will not stop to discuss, since the fact is sufficiently apparent to every person whose labors confine him to the office or store, year after year, without rest or vacation.

The injurious effects upon health of such a life being at once acknowledged and recognized, we are often asked for a remedy. "What shall I do?" The same prescription that was made by good, old Sir Isaac Walton, centuries ago, retains its efficacy and delightful pleasures, even to the present time—"Go a fysshynge!"

To the inexperienced angler, having in mind the reed pole, heavy line, and the bob-and-sinker with which he jerked, high into the air, the unsuspecting bull-head, or the less pretentious "shiner," of some neighboring pond or bayou, using for their alurement the angling worm or conventional "white grub," such advice will be unappreciated and unheeded. But, if they could have the patience to read of how *we* perform our angling, the apparatus used, the preparations for the journey, our camp building, and the jolly, congenial friends who accompany us, perhaps we could induce them to believe as do we, that no sport out of doors can equal it.

Just one month ago to-day, or June 1st, we started upon our annual vacation, to the trout streams of northern Pennsylvania, having busied ourself the week previously—and enjoying every minute of the time—in making the following preparations: We ground our ax and hatchet, filed a small hand-saw, and the cutting edge of an inch-and-a-half auger; varnished and repaired our rods; manufactured leaders and flies; oiled our reels and arranged our fly-book; overhauled our tents, and placed our camp equipage in perfect order. These we packed securely in a large, strong camp-chest, and added three long-handled frying-pans, a two quart coffee pot, a smaller tea pot, a long-handled, six quart boiling kettle, in which to boil potatoes, after which it seved for a dish-pan; one dozen tin plates, half-dozen tin cups, one dozen tin spoons, three large spoons, half-dozen knives and forks, a large, sharp, butcher's knife, a pepper and salt box. Beside of them we carefully stowed the following provisions, intended to subsist four persons four weeks, (and it did it nicely, with nothing left the day we broke camp): Twenty-five pounds of A. sugar, in a neat sugar bucket, with cover; ten pounds of coffee, four pounds of black tea, ten pounds of fat, salt pork, small sack of corn meal, one quart of table salt, small box of pepper, four quarts of beans, one peck of Bermuda onions; and, upon top of all, a small A. tent, the quarters of Fred., our cook.

In a large Saratoga trunk, we packed four comfortables, four pillows, two bed ticks, and a wall tent—nine feet square, together with a fly, twelve by fifteen feet. With this luggage, and other paraphernalia, we started by rail, for Bodines, a station on the Northern Central Railway, just sixty-two miles from this city. Leaving Elmira at 8:30 in the morning, we arrived at our destination at a quarter before eleven, when our baggage was immediately transferred to a large boat, not a hundred yards distant, upon the millpond, belonging to Squire Bodine. It required but little time to get us, bag and baggage, afloat in the Squire's big boat, for John and William, his two stout, willing boys were there to help. By noon, we had paddled up the pond to a beautiful little island, at the base of a high mountain, where, peeping out of the laurel rhododendron and ferns, is a lovely, shady bank, commanding a most enchanting view of the pond in front, and the creek and mountains on either side; and this is our annual camping ground. Here we pitched our tents—Fred's in the rear, the wall-tent next, and the large fly in front, for the parlor and for shelter in cooking during a rain. Under the fly we placed one of Dunck-lee's camping stoves, which is one of the most useful and complete affairs that we have ever used in a camping excursion. It packs in a space 12x 20 inches and contains an oven, pipe and all the cooking utensils that are needed for a company of four. For a party intending to camp further from a railway than did we, this camp stove contains everything that is needed for cooking purposes.

In the wall tent, we made two bunks of the drift wood found in the vicinity, in which our ax, hatchet, auger and nails played an important part. We covered the bunks with hemlock boughs, filled our ticks with straw from Squire Bodine's barn, paddled them to camp in the big boat, laid them upon the boughs, spread clean sheets over them, and, with the pillows and comfortables, we constructed beds upon which we slept as soundly and from which we awoke quite as much rested and refreshed as from our more pretentious ones of curled hair and elastic springs, at home.

By the middle of the afternoon, our camp was in readiness for house-keeping—beds made, camp fire constructed,—with two pieces of scrap iron, from an abandoned railroad on the hillside, placed across two timbers, upon which Fred. set his pans and pots and under which he built his fire, in preparing our meals. A box had been sunken in the bank near the creek, in which we deposited ice, bread, butter, milk and various other substantials, obtained from mother Bodine at the farm house. This we called our ice house, and served us splendidly as such, keeping our provisions and the fish we captured, fresh and sweet. In front of the tent, and between it and the camp fire, we constructed a sort of bough-house, in which we built our dining table and surrounded it with comfortable seats, made from the slabs taken from the drift-wood.

With these preparations, afterward greatly improved and added to, during our leasure moments, we spent our four week's vacation, added nearly ten pounds to our weight, an unmeasured quantity of strength to our muscles, and most refreshing rest to our somewhat burdened brain. And this is the manner in which it was done:

In the morning, early, Fred's reveille upon a large frying pan, summoned us to a breakfast of trout rolled in corn meal, and fried in a small quantity of salt pork; a splendid cup of coffee; boiled potatoes; and some piping hot flap-jacks, served with good butter and maple syrup. This, eaten in the open air, just as the sun came peeping over the mountain top, causing the beautiful clear stream on either side to sparkle as it rippled by, in seeming delight for its genial rays, while the wood-robin—the most musical of our forest songsters, the robin-redbreast, the oriole, the cat-bird and brown thrush, gave us a morning concert as delightful and charming as it was varied and novel. Eat! Gracious, who couldn't eat under such circumstances and with such surroundings? By eight o'clock our seven ounce, Conroy rods were adjusted, click reels, with light, silk lines in position, and to the slender leader is made fast our favorite flies for the day's cast; when, with feet incased in heavy, hob-nailed brogans, buckled over the flannel pants, half way to the knee; creel, over the light jacket, with landing-net attached to a large button to the collar, on our backs, the start is made for the half day's fishing, with tackle and dress that would astonish the devotee of bob and sinker.

As we journey up the stream we pass many well-known pools, note the change of channel in the creek since last year, and relate the victories achieved in landing magnificient trout, from under the various rocks and stumps which are as familiar as the faces of our friends. When we have gone far enough to occupy the time until our dining hour—two o'clock—we wade into the stream, a few rods apart, and commence casting our flies over the water, in such localities as our experience has taught us trout are apt to lie. Soon, we hear a regular war-whoop from H., way up the stream, and from the manner in which his delicate rod bends and from the appearance of an occasional splash in the water, accompanied with vigorous yells from the merry fisherman every time the trout leaps into the air, we perceive he has hooked what is technically called, a "buster." Presently we hear the merry song coming from H's vicinity, to the effect that "trout, delicious caught are," sung with such a tone of satisfaction that we at once conclude that that particular trout has been safely landed, and duly deposited in his creel.

In this manner, we wade down the stream toward our camping ground and to the delicious dinner which Fred. is sure to have in waiting for us, promply at two o'clock. Occasionally, we hear a merry song from H., then a happy refrain from S., which comes clear and melodiously through the morning air, and from which we catch an occasional sentiment to the effect that "Some love to roam," or "A gay old trout from his hole came out," indicating to us that their pleasure and success is equal to our own. As we plod along we are careful to throw our flies lightly over the pools and under the bushes that rewarded us with fine fish the season before. Should our "great dun" and "queen of the water," not entice our wary fish to the surface as plentifully as we desire, we seat ourself upon some projecting boulder in the bosom of the stream, and, while we leasurely change our flies for the "Hamlin fly," "bright fox," or imitate as nearly as may be, the natural fly upon the water, we enjoy every moment of the time as we take in the charming scene around us. Before us is the crystal creek, glistening and sparkling in the morning sun, as it ripples down a rapid or is dashed into foam against the rocks at the foot of the mountain, and then flows quietly away into the smooth and placid pool below, where the silver and golden hued naiads of the mountain streams, for which we are searching, are warily watching for their morning repast. On either side of the narrow valley, majestic and solemn looking mountains rise far, far above our heads, and are covered here with scraggly, moss-covered rocks, from which the crystal waters are dropping and sparkling in the clear, bright morning sun, like diamonds in a lady's necklace; from yonder peak the immense old hemlocks, with their dead branches and sharp pointed tops, pierce the very clouds, while the green trees upon the opposite side, give the varied tints of the hemlock, beech, birch, spruce and maple, arranging their foliage against the sky beyond, into figures grotesque or fanciful, to suit the mood in which we find ourself. With such surroundings, it matters little whether the fishing is good or bad, for one can come contented to the noon repast, with a dozen trout, a keen appetite, and a thankful spirit that he is enabled to so thoroughly enjoy nature in all her lovliness.

Our dinner, of fried onions and potatoes, boiled trout, baked johnny-cake, and a broiled steak, upon a bed of coals, over with, we lay in the cool shade of the camp, smoke our pipes, and relate the adventures of the day. Charles busied himself in constructing rustic seats, and banking up and leveling off the grounds in front of the camp, at which he is a great adept, performing the labor of two ordinary men, and enjoying himself as only a let-loose Brooklynite can. To his genius and skill the campers were indebted for many comforts and numerous well told stories, at the evening camp fire. In the evening we would throw the fly for the benefit of such old trout that would indicate their whereabouts, by a break upon the smooth surface of the pools near our camp; and many were the beauties that spread their golden sides upon the pans in our ice house, through such inadvertancy.

During our stay, we made excursions to Rock run, which empties into Lycoming creek at Ralston, a charming summer resort, with a splendid and well kept hotel, conducted by Chet. Myers, an old sportsman, assisted by his wife, who knows not only how to cook trout to a turn, but has a graceful way of making her guests comfortable and happy. This hotel is near the mouth of Rock run, which we regard as one of the most wonderful streams in our country,—far more picturesque than Watkins Glen, equally as grand and beautiful as Niagara or Trenton falls. It abounds in trout, is easy of access, and must some day become one of the popular resorts of our country. If you go there, inquire for our friend C., who spends most of the summer on this run, and is always ready to accompany any real lover of nature, upon an excursion to its beautiful falls, cascades and glens, and who will at noontime teach you how to cook a trout, rolled in a buttered paper, and thrown in the hot coals for ten minutes, that will melt in your mouth and rival in delicious flavor, any morsel of which you ever have partaken.

Tim Gray's run, two miles below our camp, is another famous resort for fishermen. We visited this stream, several times, but found it nearly exhausted of fish, as are indeed, most of the streams in this neighborhood. Still, a good angler, one who can throw the fly skillfully, can catch from twenty to thirty, good fish, in a day, under favorable condition of stream and weather. Hiring squire Bodine's team, we have frequently driven six miles up the stream and then cast the fly until we reach the saw mill, about two miles from the mouth of the creek. Here, Charles and ourself would lunch, upon the good cream and light bread furnished by Mrs. Gray, while we listened to the hunting and fishing exploits of old Tim Gray, as related by his worthy son and successor, George. To hear of the deer and bear the old man captured, and the tremendous fish he used to land upon the banks of this famous creek, made us wish our taste for angling had been developed forty years ago, when old Tim Gray was the sole inhabitant along the creek which bears his name.

George, not only told us many stories, but gave us many practical hints in angling, that were new even to an old fellow like ourself.

Fishing down this stream to its mouth, we killed several fine fish, and hooked one which we estimated to weigh at least one and-a-half pounds, in an old eel wear in the main creek. H. had hooked and lost him the day before, and indicated to us where the old chap lay, but by reason of swift water and an old log under which he would take refuge, he parted company with us almost as soon as we hooked him. We had him twice after this, and lead him about by the nose for several minutes one day, but up to this writing, he continues to remain under that log and "goes for" every confounded fly, artificial or natural, that floats down that rapid to his lair. In order to relieve the minds of any piscatorially inclined, this is to give notice, that we *will* capture that trout; for, before these lines are read, we shall have thrown another over the rapid where lies our charmer, when we hope to have the satisfaction of relating that he has been safely brought to our creel.

Well, so we passed our days, until the fish became so scarce as to induce H. and S. to leave us for more bountifully stocked waters, while Charles and ourself enjoyed the air, the food, the bathing, and the rest and quietness of our camp life. Once a bevy of ladies and gentlemen from the city,—our friends, made an excursion to our camp, praised everything they saw, and *eat* everything they praised; spent a day rambling in the woods and an evening by the camp-fire and expressed a desire to become campers and anglers too. Several times, acquaintances from Ralston visited us, with whom we had pleasant, jolly times; and so the days flew by, until the time for our departure arrived, when we broke camp, and started for home, heavier in flesh, stouter in muscle, rested in mind, strong, and eager again for our professional duties. Our friends testify to our improved appearance, while we are conscious that our four weeks in the woods, were pleasureably and profitably spent, and we think our readers must agree with us that such an excursion is incalculably better, both for body and purse, than to spend the same length of time at a fashionable watering place, in the vain endeavor to rest and restore one's exhausted vitality. And that's the way we go angling. Do you think you could enjoy it? If so, try it, and our word for it, you will seek no other recreation, nor find no keener sport.

